# Activation of TRPV1 channel by dietary capsaicin improves visceral fat remodeling through connexin43-mediated Ca^2+^ Influx

**DOI:** 10.1186/s12933-015-0183-6

**Published:** 2015-02-13

**Authors:** Jian Chen, Li Li, Yingsha Li, Xia Liang, Qianqian Sun, Hao Yu, Jian Zhong, Yinxing Ni, Jing Chen, Zhigang Zhao, Peng Gao, Bin Wang, Daoyan Liu, Zhiming Zhu, Zhencheng Yan

**Affiliations:** Center for Hypertension and Metabolic Diseases, Department of Hypertension and Endocrinology, Daping Hospital, Third Military Medical University, Chongqing Institute of Hypertension, Chongqing, 400042 China

**Keywords:** TRPV1, Cx43, Obesity, Ca^2+^, Visceral fat remodeling

## Abstract

**Background:**

The prevalence of obesity has dramatically increased worldwide and has attracted rising attention, but the mechanism is still unclear. Previous studies revealed that transient receptor potential vanilloid 1 (TRPV1) channels take part in weight loss by enhancing intracellular Ca^2+^ levels. However, the potential mechanism of the effect of dietary capsaicin on obesity is not completely understood. Ca^2+^ transfer induced by connexin43 (Cx43) molecules between coupled cells takes part in adipocyte differentiation. Whether TRPV1-evoked alterations in Cx43-mediated adipocyte-to-adipocyte communication play a role in obesity is unknown.

**Materials and methods:**

We investigated whether Cx43 participated in TRPV1-mediated adipocyte lipolysis in cultured 3T3-L1 preadipocytes and visceral adipose tissues from humans and wild-type (WT) and TRPV1-deficient (TRPV1^-/-^) mice.

**Results:**

TRPV1 and Cx43 co-expressed in mesenteric adipose tissue. TRPV1 activation by capsaicin increased the influx of Ca^2+^ in 3T3-L1 preadipocytes and promoted cell lipolysis, as shown by Oil-red O staining. These effects were deficient when capsazepine, a TRPV1 antagonist, and 18 alpha-glycyrrhetinic acid (18α-GA), a gap-junction inhibitor, were administered. Long-term chronic dietary capsaicin reduced the weights of perirenal, mesenteric and testicular adipose tissues in WT mice fed a high-fat diet. Capsaicin increased the expression levels of p-CaM, Cx43, CaMKII, PPARδ and HSL in mesenteric adipose tissues from WT mice fed a high-fat diet, db/db mice, as well as obese humans, but these effects of capsaicin were absent in TRPV1^-/-^ mice. Long-term chronic dietary capsaicin decreased the body weights and serum lipids of WT mice, but not TRPV1^-/-^ mice, fed a high-fat diet.

**Conclusion:**

This study demonstrated that capsaicin activation of TRPV1-evoked increased Ca^2+^ influx in Cx43-mediated adipocyte-to-adipocyte communication promotes lipolysis in both vitro and vivo. TRPV1 activation by dietary capsaicin improves visceral fat remodeling through the up-regulation of Cx43.

## Background

Obesity is the result of sustained mismatches that favor caloric intake over caloric expenditure. The prevalence of obesity during the last few years has dramatically increased worldwide [1]. In addition to many other disorders, obesity greatly increases the risk of developing a metabolic syndrome and is associated with decreased life expectancy and increased health costs [2]. For these reasons, obesity has attracted increasing attention across multiple scientific disciplines. Although the adipose tissue of obese animals and humans is increased at both subcutaneous and visceral sites, visceral fat is responsible for the metabolic consequences of obesity [3]. The reasons for this effect are unclear. Visceral fat mass is closely related to the metabolic consequences of obesity; visceral adipose tissue depots are key correlates of the metabolic abnormalities associated with being overweight/obese, including inflammation. The release of cytokines, particularly IL-6, from fat cells may stimulate the proinflammatory state that characterizes obesity. The increased secretion of prothrombin activator inhibitor-1 from fat cells may play a role in the procoagulant state of obesity and, along with changes in endothelial function, may be responsible for the increased risk of cardiovascular disease and hypertension [4-6].

Capsaicin, the major pungent ingredient in hot peppers, is a potent agonist for the transient receptor potential vanilloid 1 (TRPV1) channel, also called the capsaicin receptor. TRPV1 is a non-selective cation channel with a preference for cations; it transduces signals for the sensations of noxious heat and pain [7]. TRPV1 is highly expressed in sensory neurons and in vasculature, adipose, and liver tissues [8-10]. The earlier studies from human showed an increase in diet-induced-thermogenesis and lipid oxidation when high fat diet with capsaicin [11,12]. But studies of TRPV1 in weight loss present mixed results and the controversies. Marshall N J, et al, their results do not support the concept that TRPV1 plays a major role in influencing weight gain [13]. Gram DX, et al found that capsaicin had no effect on Zucker diabetic fatty (ZDF) animals weight loss, and the terminal body weight of the capsaicin-treated rats was significantly higher than that of the vehicle-treated ones [14]. An increase in energy expenditure after capsaicin in take was supported by studies by various investigators [15-17]. Some studies revealed a role for TRPV1 in promoting fat accumulation and weight gain because long-term TRPV1 antagonists or agonists administration can promote TRPV1 desensitization [18,19]. Our previous studies detected TRPV1 channels in 3T3-L1-preadipocytes and visceral adipose tissue from mice and humans. Activation of TRPV1 channels by capsaicin prevented adipogenesis and obesity [20] and nonalcoholic fatty liver disease [21]. However, the potential mechanism of the effect of dietary capsaicin on visceral fat accumulation and remodeling is not completely understood.

Gap-junctional communication (GJC) plays critical roles in cell growth and differentiation. Gap junctions allow the transfer of ions and small molecules between coupled cells and enable individual cells to communicate with their neighbors and match the immediate needs of coupled cells within a cluster [22]. The major component of gap junctions is connexin43 (Cx43), which exists in almost all tissues and in 3T3-L1 cells; this molecule allows for cell-to-cell communication. Intracellular Ca^2+^ is one of the important signals in adipogenesis and regulates Cx43 function in adipocytes. The expression of Cx43 is down-regulated at the transcriptional level during adipogenesis in the H-1/A cell line, a marrow stromal cell line that differentiates into adipocytes [23]. More recently, Yanagiya et al. reported that functional gap junctions are required for progression through a specific early stage of the 3T3-L1 preadipocyte differentiation program. However, the role of Cx43 is different during the later stages of adipogenesis, particularly during adipocyte maturation. Inhibiting Cx43 degradation or constitutively over-expressing Cx43 blocks adipocyte differentiation via increases in intracellular ATP and [Ca^2+^] [24,25]. Therefore, GJC plays some important roles in adipocyte differentiation and lipid metabolism. TRPV1-evoked alterations in Cx43-mediated adipocyte-to-adipocyte communication play an important role in obesity. Here, we show that the activation of TRPV1 by dietary capsaicin affects visceral fat accumulation and remodeling through a Cx43-mediated cell-to-cell intracellular [Ca^2+^] increase.

## Materials and methods

### Animal treatment and experimental procedures

All mice (C57BL/6 WT mice, TRPV1^-/-^ mice and db/db mice) were purchased from Jackson Laboratory (Bar Harbor, Maine, USA). TRPV1^-/-^ mice which TRPV1 gene was knocked out in the whole body, not conditioned knockout we used in the experiments. WT and TRPV1^-/-^ male mice were randomized into three groups respectively (n = 15, 5 mice/cage) at 4-6 weeks of age, one group received standard laboratory chow, one group received high-fat diet, one group received high-fat diet plus 0.01% capsaicin. The high-fat diet supplied 49% of the calories as fat and 30% of the calories as carbohydrate. Db/db male mice were randomized into two groups respectively (n = 6, 6 mice/cage) at 4-6 weeks of age, one group received standard laboratory chow, the other group received standard laboratory chow plus 0.01% capsaicin. The standard laboratory chow diet provided 10% of the calories as fat and 68% as carbohydrate. All mice were housed in colony cages with a 12-hour light/12-hour dark cycle with free access to food and water for 5 months, body weight was measured every two weeks. At the end of 5 months, blood glucose was measured. Visceral fat was removed and weighted. Serum lipids were analyzed using routine techniques. All of the experiment procedures were performed in accordance with protocols approved by the Institutional Animal Care and Research Advisory Committee.

### Subjects Characteristics

We recruited the obese male subjects and those were classified if waist circumference was more than 90 cm according to the Asian criteria of WHO Regional Office for the Western Pacific/International Association for the Study of Obesity/International Obesity Task Force. Age, body mass index, waist circumference were obtained. Visceral fat tissues were obtained from patients during regular scheduled cholecystectomy. Cholecystectomy had to be performed because of symptomatic gallstones. The protocol was approved by the local Ethics Committee. All subjects gave written informed consent.

### Histopathologic examination

The adipose tissue was cleaned with saline and weighted. Visceral fats from WT mice and human were observed with frost slice techniques and stained with anti-TRPV1 or Cx43 antibodies [20]. The TRPV1 and Cx43 in isolated 3T3-L1 preadipocytes or adipocytes primary cultured from visceral adipose tissue were identified by immunofluorescence stain. 3T3-L1 preadipocytes were cultured, fixed, and stained with the lipophilic dye oil-red O (Sigma-Aldrich). Red staining showed lipid droplets in the cytoplasm, indicating the number of lipid droplets in 3T3-L1 preadipocytes according to established techniques [26].

### Cell culture and adipocyte differentiation assay

Murine 3T3-L1 preadipocytes were cultured and maintained in Dulbecco’s modified Eagle’s medium supplemented with 10% fetal calf serum (Hyclone) containing 100 μg/ml penicillin and 100 μg/ml streptomycin (GIBCO). Cells were plated and grown until 2 days post-confluence. Differentiation was then induced (day 0) by changing the medium to Dulbecco’s modified Eagle’s medium supplemented with 10% fetal calf serum, 0.5 mmol/L 3-isobutyl-1-methylxanthine, 1 μmol/L dexarmethasone, and 10 μg/ml insulin (Sigma-Aldrich). After 2 days the medium was replaced with the medium containing only 10 μg/ml insulin [27]. To confirm the functional properties of TRPV1 channels and Cx43 gap-junction on adipogenesis of 3T3-L1 preadipocytes, on the 8^th^ day after differentiation, the TRPV1 agonist capsaicin (1 μmol/L), or TRPV1 antagonist capsazepine (1 μmol/L), or inhibitor of Cx43 gap-junction 18 alpha-glycyrrhetinic acid (18α-GA, 150 μmol/L), (Sigma-Aldrich) were added to the medium for 24 hours as indicated [28]. Free fatty acids (FFA) (oleate/palmitate [PA], 2:1; Kelong Chemical Reagent Factory, Chengdu, China) were mixed with FFA-free bovine serum albumin, and the mixture was added to the medium to a final concentration of 1 mmol/L [21]. Intracellular accumulation of lipid droplets in 3T3-L1 preadipocytes were stained with Oil Red O (0.5 g in 100 ml isopropanol) at the day 3, 5, and 8 with or without differentiation.

### Intracellular free calcium measurement

3T3-L1 cells grown on glass cover slips were loaded with Ca^2+^ indicator Fura-2 (2 μmol/L, Invitrogen, Paisley, UK) and 0.025% Pluronic F-127 in a physiological saline solution for 40 min at room temperature in the dark. [Ca^2+^]_*i*_ was measured using a fluorescent plate reader (Varioskan Flash, Thermo) at 510 nm emission, with excitation wavelengths of 340 nm and 380 nm. The changes in [Ca^2+^]_*i*_ were calculated from the ratios of transient increases in fluorescence intensity at 340 nm and 380 nm [8].

### Fluorescence Recovery after Photo bleaching (FRAP)

All fluorescent dyes emit light of one wave length (e.g. green) after they have absorbed light of another wave length (e.g. blue). However, if a very high intensity blue light is delivered to the dye, the dye will “photobleach” meaning that the high intensity light has rendered the dye unable to fluoresce. This phenomenon has lead to an interesting method called Fluorescence Recovery After Photobleaching (FRAP). The idea behind this method is to use FRAP to measure the ability of a molecule to move around over time. The percent recovery uses the formula: (Y/ X) × 100 = % recovery. In the diagram, the percentage of fluorescence lost due to photo bleaching is X and the amount of fluorescence that returned to the bleached area is Y. FRAP evaluates the functional effect of gap junctions for intercellular communication between adjacent cells by dye coupling studies, in which photobleaching of cells loaded with a membrane-permeable fluorescence dye, 5, 6-carboxyfluorescein diacetate (5, 6-CFDA, Invitrogen Corp, Carlsbad, CA, USA), resulted in rapid recovery of fluorescence into the photobleached cell, within 10 min postbleaching [29,30]. After treatment, cells were rinsed twice with 1 ml of Hank buffer and incubated with 5, 6-CFDA (8 μg/mL in DMEM) at 37°C for 15 minutes, and quantitative imaging was excited at 488 nm by an argon ion laser and captured through a gating at 530/30 nm. The analyzed fluorescence recovery index is expressed as: R = (IR-I0)/(Ii-I0) × 100%. Fluorescence recovery was normalized with unbleached control to compensate fluorescence lost during the experiment [31].

### Immunoblotting analysis

Immunoblotting of TRPV1, Cx43, p-CaM, CaMKII, PPARδ, HSL, β-actin and GAPDH were performed using standard techniques for adipose tissue and mature adipose cells. Primary antibody for TRPV1 was purchased from Alomone, Israel and other primary antibodies were from Santa Cruz Biotechnology (Santa Cruz, CA, USA). After incubation with the secondary antibodies for 1 h, the proteins were detected by enhanced chemiluminescence and quantified using a Gel Doc 2000 Imager (Bio-Rad).

### Measurement of triglyceride and free fatty acid in cells

Total lipids were extracted from 3T3-L1 preadipocytes using a chloroform-methanol (2:1, vol/vol) mixture. Triglyceride and free fatty acid levels were quantified using ELISA kit (Applygen Technologies Inc., China) according to the manufacturer’s instructions. Cell extracts were collected and centrifuged at the speed of 10000 rpm for 15 min to obtain the supernatant. Then 100 μl supernatant and 50 μl enzyme conjugate were added to the provided 96-well plate for 1 h in 37°C while standard curve was made in the same plate. Afterwards, each well was washed with the diluted washing solution for 5 times, thoroughly pat dry with absorbent paper. Then 50 μl substrate A and 50 μl substrate B were successively added and the plate was incubated in 37°C for 15 min. At last, stop solution 50 μl was then added to each well and the OD450 values were measured with the Varioskan Flash, Thermo.

### Statistical analysis

The data were expressed as the means ± SEM from three to 3-15 independent experiments or mice. Comparisons between groups were analyzed using the Student’s *t* test or one-way ANOVA with Bonferroni’s multiple comparison post hoc test (GraphPad Prism; La Jolla, CA, USA). Two-tailed *p* values less than 0.05 were considered to indicate statistical significance.

## Results

### Functional TRPV1 co-expressed with Cx43 in mesenteric adipose tissues and 3T3-L1 preadipocytes

**Figure 1:**
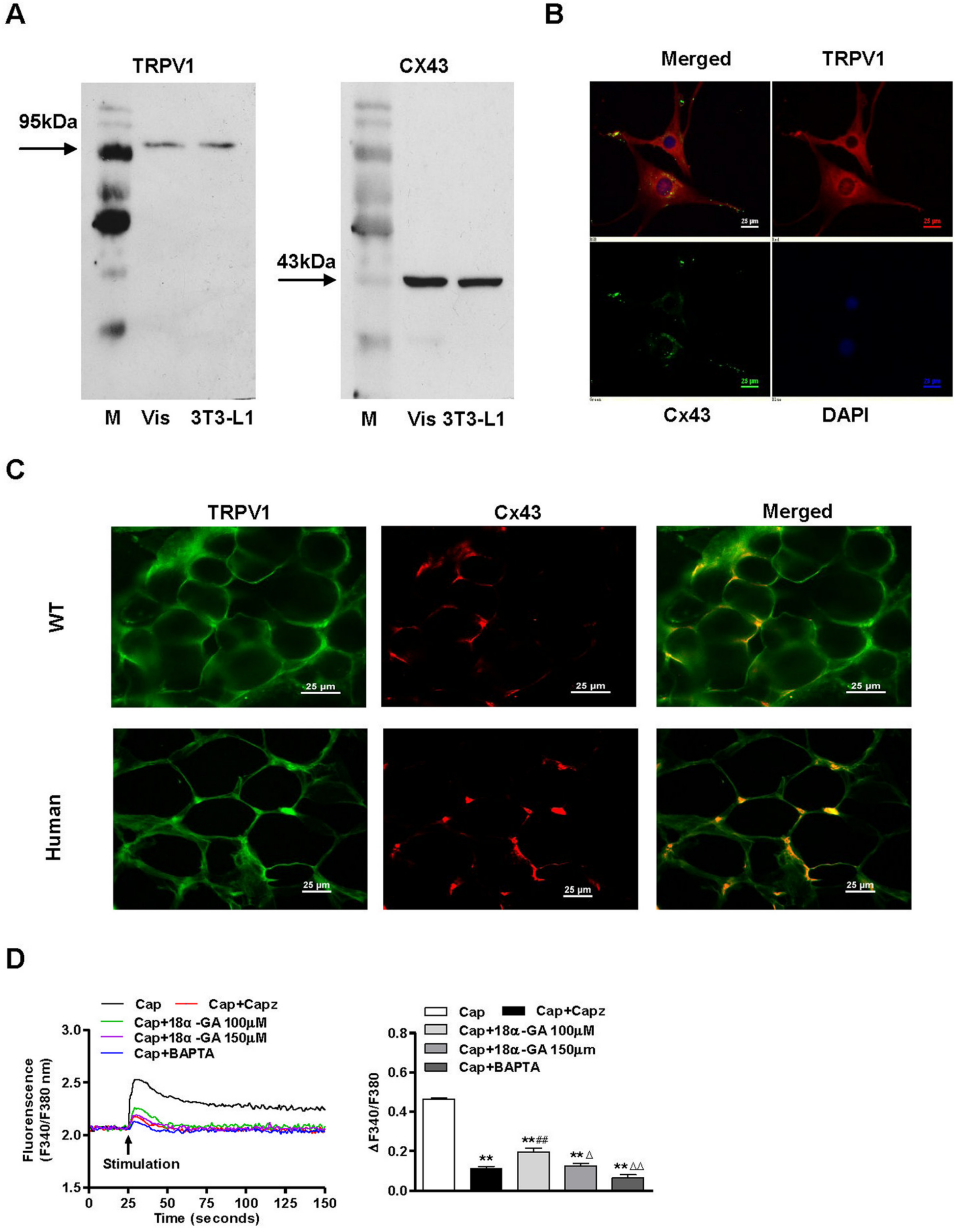
**Cx43 affects TRPV1-mediated Ca** ^**2+**^ **influx in adipocytes. A**. TRPV1 and Cx43 were detected in mesenteric adipose tissue (Vis) and 3T3-L1 preadipocytes (3T3-L1). M indicates the marker. **B** and **C**. Immunofluorescence demonstrated specific staining for co-expressed TRPV1 and Cx43 in the cell-cell connecting portions in primary cultured human visceral adipocytes **B** and visceral adipose tissues from both wild-type mice (WT) and humans **C**. The green fluorescence indicates Cx43. The red fluorescence indicates TRPV1. Nuclei in all groups were stained in blue with DAPI. The images were collected using a Nikon TE2000-U inverted fluorescence microscope and are representative of 3 separate experiments. The scale bar indicates 25 μm. **D**. Representative curves (left panel) show [Ca^2+^]_*i*_ changes in 3T3-L1 preadipocytes acutely stimulated with capsaicin (Cap, 1 μmol/L), capsaicin with the TRPV1 antagonist capsazepine (Cap + Capz, 1 μmol/L), the Cx43 inhibitor, 18α-glycyrrhetinic acid (Cap + 18α-GA, 100 or 150 μmol/L), or the intracellular Ca^2+^ chelator, BAPTA-AM (10 μmol/L). The summary data (right panel) show the maximal stimulated changes of [Ca^2+^]_*i*_ (25-150 s) from the baseline (0-25 s). Values were expressed as the mean ± SEM; n = 4 per group. **P < 0.01 vs. Cap; ^##^P < 0.01 vs. Cap + Capz; ^Δ^P < 0.05, ^ΔΔ^P < 0.01 vs. Cap + 18α-GA 100 μM.

First, we detected TRPV1 and Cx43 in visceral adipose tissues from mice and 3T3-L1 preadipocytes by immunoblotting. The immunoblots confirmed the TRPV1 and Cx43 molecular weights at 95 kDa and 43 kDa, respectively, and the antibodies identified TRPV1 and Cx43 in both mesenteric adipose tissues and 3T3-L1 preadipocytes (Figure 1A). Immunofluorescence demonstrated specific staining for co-expressed TRPV1 and Cx43 in the cell-cell connecting portions in primary cultured human visceral adipocytes and visceral adipose tissues from both wild-type mice (WT) and humans. The green fluorescence indicates Cx43. The red fluorescence indicates TRPV1. Nuclei in all groups were stained in blue with DAPI (Figure 1B and C). Cx43 is a gap junction protein, and 18α-GA is the gap-junction inhibitor widely used to inhibit gap junctional communication that is likely to enter cells and modify protein kinases that regulate Cx43 [32]. Capsazepine is a TRPV1 antagonist [33]. TRPV1 belongs to the family of nonselective cation channels that display high Ca^2+^ permeability. Acute exposure to capsaicin, a TRPV1-specific agonist, stimulated an increase in the cytosolic free calcium concentration ([Ca^2+^]_*i*_) in cultured 3T3-L1 preadipocytes. According to a previous study, calcium ions are most likely to travel through GJs, and the differential Ca^2+^ uptake into intracellular stores at high and low concentrations is influenced by slight time delays of Ca^2+^ traveling through the GJs. TRPV1-elicited Cx43 gap-junction assay showed that response of Ca^2+^ to capsaicin was completely inhibited by 150 μM 18α-GA in Cx43-expressing HeLa cells [34]. We found that the capsaicin-induced calcium increase could be inhibited by 18α-GA at a concentration of 100 μmol/L, whereas the maximum effect was obtained at 150 μmol/L in 3T3-L1 preadipocytes. We also showed that 18α-GA dose-dependently inhibited calcium influx by capsaicin-induced activation of TRPV1, and that 18α-GA (150 μM) could completely block the increased calcium influx by capsaicin, as capsazepine effect in 3T3-L1 preadipocytes (Figure 1D). These results suggest that functional TRPV1 and Cx43 exist in adipose tissues and cells, both in humans and mice. Inhibition of TRPV1 and Cx43 reduces the increased free cytosolic calcium concentration ([Ca^2+^]_*i*_) in 3T3-L1 preadipocytes.

### Inhibition of Cx43 reduces the cytosolic calcium increase induced by TRPV1 activation and prevents adipolysis in 3 T3–L1 preadipocytes

**Figure 2:**
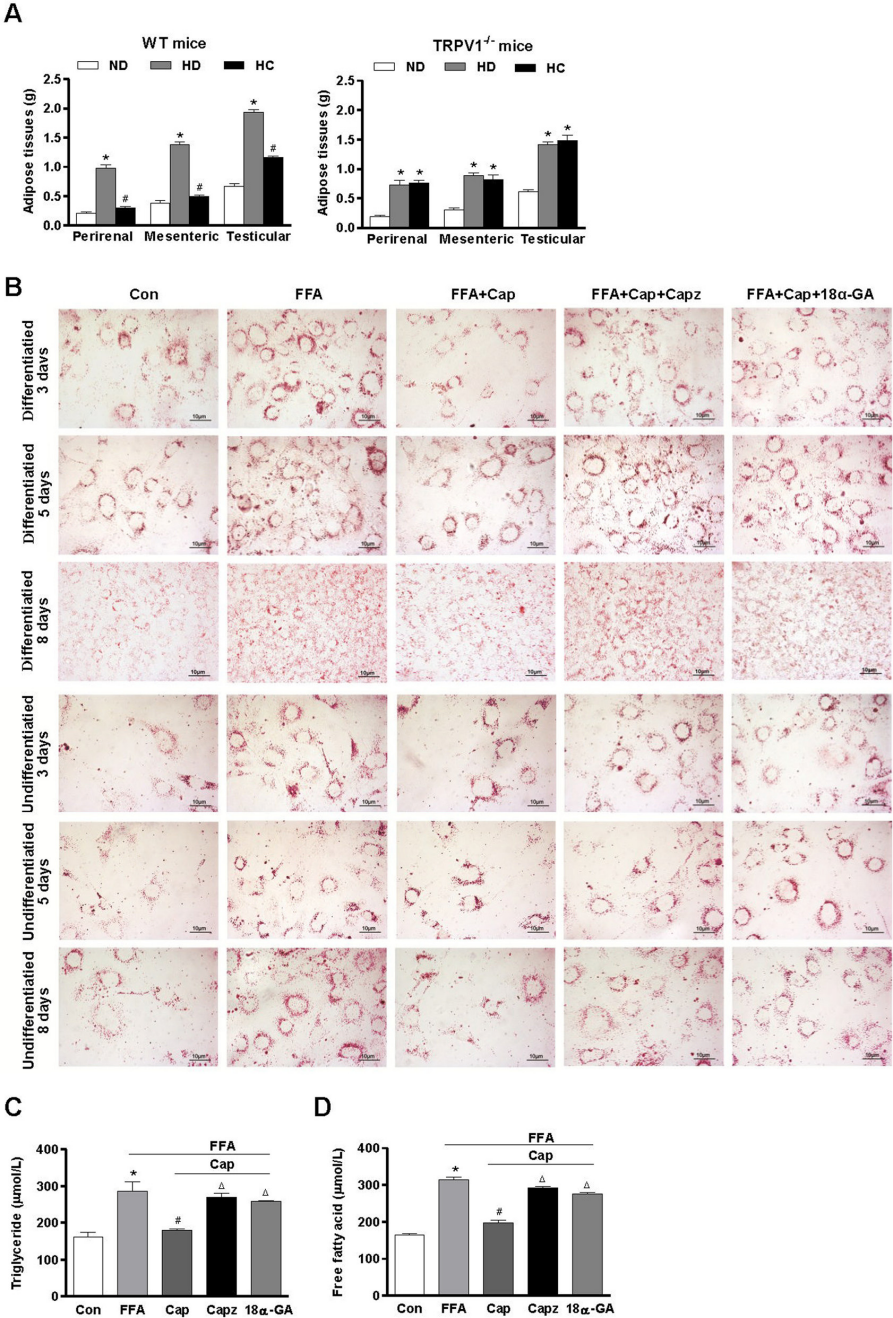
**TRPV1 activation promotes lipolysis both in mesenteric adipose tissue and** 3T3-L1 **preadipocytes. A**. The weights of mesenteric, perirenal and testicular adipose tissues from WT and TRPV1^−/−^ mice fed a normal diet (ND), a high-fat diet (HD), or a high-fat plus capsaicin diet (HC). *P < 0.05 vs. ND, ^#^P < 0.05 vs. HD. Values are expressed as the mean ± SEM, n = 6. **B**. Lipid droplets were visualized by Oil red O staining of 3T3-L1 preadipocytes on days 3, 5, and 8, with or without differentiation. The cells were exposed to a mixture of 1 mmol/L long-chain free fatty acids (FFA) in the presence of 1 μmol/L capsaicin (FFA + Cap), capsaicin plus 1 μmol/L capsazepine (FFA + Cap + Capz) or 150 μmol/L 18α-GA (FFA + Cap + 18α-GA) for 24 h. Red spots indicate lipid droplets in the cells. Images are representative of 3 separate experiments. The scale bar indicates 10 μm. **C** and **D**. Triglyceride and free fatty acid levels of 3T3-L1 preadipocytes after treatment with free fatty acids (FFA), FFA + capsaicin (Cap), FFA + capsaicin + capsazepine (Capz) or FFA + capsaicin + 18α-GA (18α-GA, 150 μmol/L) for 24 h. *P < 0.05 vs Con, ^#^P < 0.05 vs FFA, ^Δ^P < 0.05 vs Cap. Values are expressed as the mean ± SEM; n = 3 per group.

Our previous study showes that the administration of capsaicin prevents obesity in male WT mice but not in TRPV1^−/−^ mice fed a high-fat diet [20]. In the present study, we found that dietary capsaicin also reduced the weights of perirenal, mesenteric and testicular adipose tissues in WT mice but not TRPV1^−/−^ mice fed a high-fat diet (Figure 2A). Based on the previous findings by Zhang et al., TRPV1 activation by capsaicin increases intracellular calcium concentrations and prevents adipogenesis in 3T3-L1 preadipocytes. We evaluated the effects of Cx43 on lipid deposition which regulated by TRPV1 in 3T3-L1 preadipocytes. Adipogenesis was induced in 3T3-L1 preadipocytes, as described in our previous study [20]. Lipid droplets were visualized by Oil red O-staining in 3 T3–L1 preadipocytes on days 3, 5, and 8, with or without differentiation, in the absence (control condition) and presence of capsaicin (TRPV1 agonist), capsaicin plus 18α-GA (Cx43 gap-junction inhibitor), or capsazepine (TRPV1 antagonist) [24,25]. Maturation of the 3T3-L1 preadipocytes was induced by 3-isobutyl-1-methylxanthine, dexamethasone, and insulin, as recommended. Free fatty acids (FFAs) increased the number of lipid droplets after maturation was induced. Capsaicin, the TRPV1 agonist (final concentration, 1 μmol/L), reduced the amount of lipid droplets in 3T3-L1 preadipocytes. Furthermore, determination of cellular triglyceride levels confirmed that capsaicin prevented adipogenesis, whereas the administration of capsazepine or 18α-GA did not prevent adipogenesis in 3T3-L1 preadipocytes. The maximum effect of 18α-GA was obtained at a concentration of 150 μmol/L in 3T3-L1 preadipocytes (Figure 2B). We found that capsaicin induced an increase in the intracellular calcium levels and a decrease in triglyceride levels, which could be inhibited by capsazepine, a TRPV1 antagonist, at a concentration of 1 μmol/L, or 18α-GA, a Cx43 gap-junction inhibitor, at a concentration of 100 μmol/L; however, the maximum effect of 18α-GA was obtained at 150 μmol/L in 3T3-L1 preadipocytes. The accumulations of triglyceride and free fatty acids did not significantly differ between FFA, FFA + Cap plus Capz and FFA + Cap plus 18α-GA groups (Figure 2C and D). Thus, we found that the administration of FFAs significantly increased the intracellular lipid droplets in 3T3-L1 preadipocytes, whereas capsaicin led to reduced intracellular lipid droplets. In contrast, the antagonism of TRPV1 or the inhibition of Cx43 abolished the effect of capsaicin on the adipolysis of preadipocytes. Based on these experiments, it was concluded that the capsaicin-induced cytosolic calcium increase through TRPV1 channels promoted adipolysis associated with Cx43-mediated Ca^2+^ influx.

### TRPV1 promotes lipolysis of 3T3-L1 preadipocytes by regulating Cx43-mediated intracellular calcium levels

**Figure 3:**
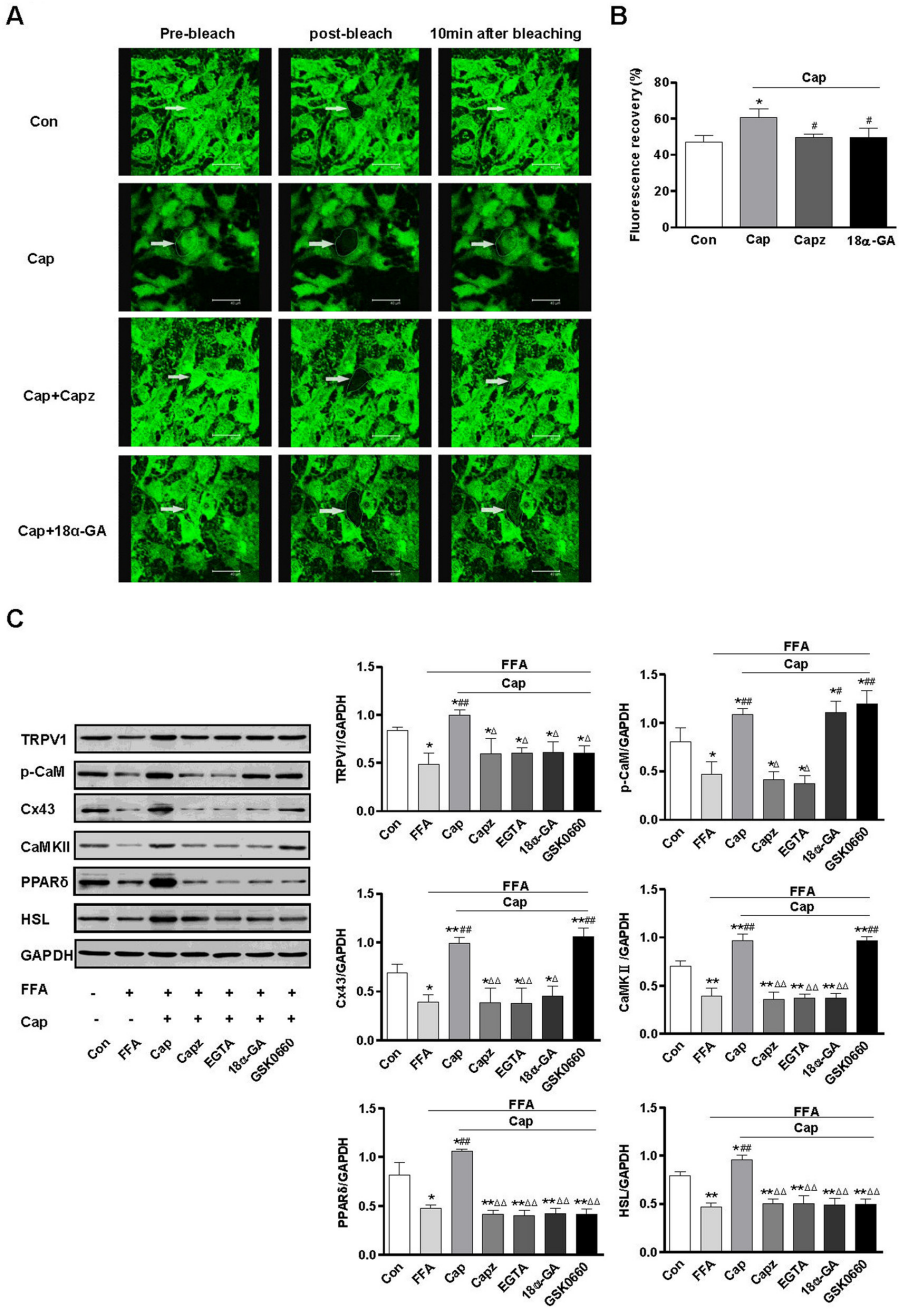
**TRPV1 promotes lipolysis of** **3T3-L1** **preadipocytes through the regulation of Cx43-mediated intracellular calcium levels. A**. Images indicate the Fluorescence Recovery After Photobleaching (FRAP), after treatment with capsaicin (Cap), capsaicin + capsazepine (Capz) or capsaicin + 18α-GA (18α-GA) for 24 h, used to measure the dynamics of Cx43 mobility. **B** The ratio of fluorescence recovery inside the bleached regions. *P < 0.05 vs Con, ^#^P < 0.05 vs Cap. The values were expressed as the mean ± SEM from 3 separate experiments. **C**. Immunoblots of TRPV1, p-CaM, Cx43, CaMKII, PPARδ, and HSL in 3T3-L1 preadipocytes treated with free fatty acids (FFA), FFA + capsaicin (Cap), FFA + capsaicin + capsazepine (Capz), FFA + capsaicin + EGTA (EGTA), FFA + capsaicin + 18α-GA (18α-GA) or FFA + capsaicin + GSK0660 (GSK0660, PPARδ inhibitor, 10 μmol/L) for 24 h. *P < 0.05, **P < 0.01 vs Con; ^#^P < 0.05, ^##^P < 0.01vs FFA; ^Δ^P < 0.05, ^ΔΔ^P < 0.01 vs Cap. The densitometric values of protein expression levels were all normalized to GAPDH. Values are expressed as the mean ± SEM; n = 3 per group.

Fluorescence Recovery After Photobleaching is used to measure the dynamics of Cx43 mobility. We used the gap-FRAP method to study the effect of TRPV1 on Cx43 in 3T3-L1 preadipocytes. The results showed that the inhibition of both TRPV1 and Cx43 induced few changes outside and little recovery of fluorescence inside the bleached regions in 3T3-L1 preadipocytes treated with both Capsazepine and 18α-GA, which damaged the dynamics of Cx43 mobility (Figure 3A and B). The 3T3-L1 preadipocytes were treated with free fatty acids (FFA), FFA + capsaicin (Cap), FFA + capsaicin plus TRPV1 antagonist capsazepine (Capz), FFA + capsaicin + ethyleneglycol-bis (beta-aminoethylether)-N, N’-tetraacetic acid (EGTA, calcium chelator), FFA + capsaicin + 18α-GA (18α-GA, 150 μmol/L) or FFA + capsaicin plus PPARδ inhibitor GSK0660 (10 μmol/L) for 24 h. The drug intervention showed that TRPV1 activation by capsaicin led to the up-regulation of Cx43, CaMKII, PPARδ and HSL. Inhibition of TRPV1 or Cx43 by capsazepine or 18α-GA and exposure to EGTA mediated the down-regulation of Cx43, CaMKII, PPARδ and HSL in 3T3-L1 preadipocytes (Figure 3C). These results indicated that TRPV1 activation by capsaicin increased intracellular calcium associated with Cx43 function and promoted lipolysis in 3T3-L1 preadipocytes.

### TRPV1 activation by capsaicin increases Cx43 mediated lipolysis of visceral adipose tissue in humans and mice

**Figure 4:**
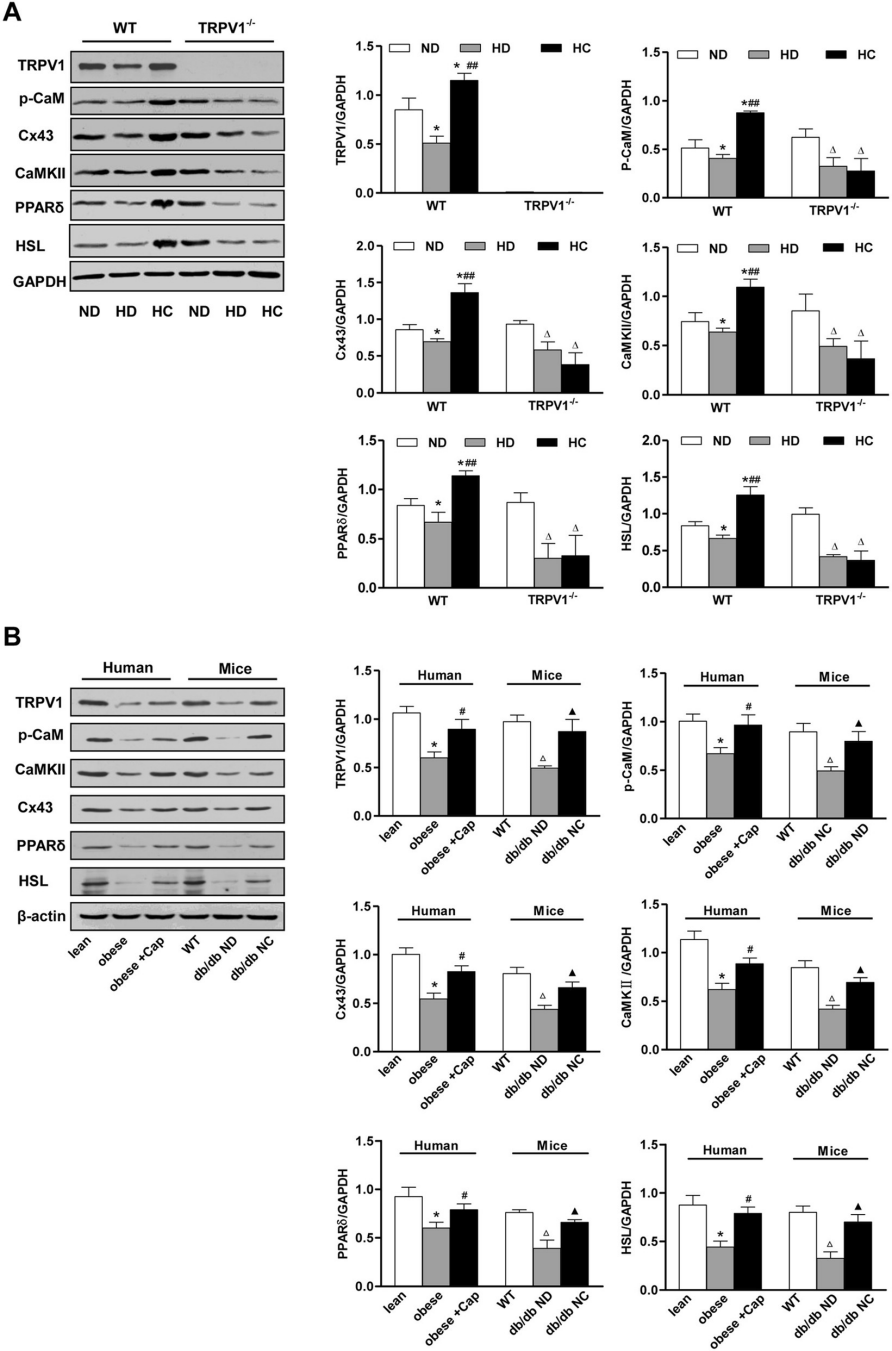
**TRPV1 activation by dietary capsaicin increases Cx43-mediated lipolysis of mesenteric adipose tissues in mice and humans. A**. Immunoblots of TRPV1, p-CaM, Cx43, CaMKII, PPARδ, and HSL in mesenteric adipose tissues from WT and TRPV1^-/-^ mice fed a ND, HD and HC. *P < 0.05 vs. WT ND, ^##^P < 0.01 vs. WT HD, ^Δ^P < 0.05 vs. TRPV1^-/-^ ND. **B**. Immunoblots of TRPV1, p-CaM, Cx43, CaMKII, PPARδ, and HSL in visceral adipose tissues from humans and WT and db/db mice. Obese + Cap indicates that visceral adipose tissues from obese humans were treated with capsaicin for 24 hours. Db/db mice were fed a standard laboratory chow (db/db ND) or standard laboratory chow plus 0.01% capsaicin (db/db NC). *P < 0.05 vs lean people, ^#^P < 0.05 vs obese people, ^Δ^P < 0.05 vs WT, ^▲^P < 0.05 vs db/db ND. The densitometric values of protein expression levels were all normalized to GAPDH or β-actin. Values are expressed as the mean ± SEM for 3 mice or people.

We further studied the effects of chronic dietary capsaicin on p-CaM, Cx43, CaMKII, PPARδ and HSL in visceral adipose tissue from WT mice or TRPV1^−/−^ mice fed a high-fat diet and mesenteric adipose tissue from obese or lean human male subjects. The results showed that, compared with a normal diet, a long-term high-fat diet significantly reduced the protein expression levels of p-CaM, Cx43, CaMKII, PPARδ and HSL in mesenteric adipose tissues from WT mice, but that these effects were reversed by chronic dietary capsaicin. In contrast, the effects of dietary capsaicin on the up-regulation of p-CaM, Cx43, CaMKII, PPARδ and HSL in visceral adipose tissue were absent in TRPV1^−/−^ mice fed a high-fat diet plus capsaicin (Figure 4A). Furthermore, we also confirmed that the expression levels of TRPV1, p-CaM, Cx43, CaMKII, PPARδ and HSL in mesenteric adipose tissue from obese human male subjects were significantly decreased compared with age-matched lean male subjects. The protein expression levels of TRPV1, p-CaM, Cx43, CaMKII, PPARδ and HSL were significantly decreased in the mesenteric adipose tissue from obese male subjects, but were reversed after the adipose tissue was treated with capsaicin for 24 h (Figure 4B). The expression levels of TRPV1, p-CaM, Cx43, CaMKII, PPARδ and HSL in mesenteric adipose tissue from db/db mice were decreased compared with those from WT mice fed a normal diet (ND). However, long term dietary capsaicin significantly increased the expression levels of TRPV1, p-CaM, Cx43, CaMKII, PPARδ and HSL in visceral adipose tissue from db/db mice compared with db/db ND group mice (Figure 4B). These results further confirm that TRPV1 activation by chronic capsaicin up-regulates p-CaM, Cx43, CaMKII, PPARδ and HSL in visceral adipose tissues from both humans and mice.

### TRPV1 activation by dietary capsaicin improves high-fat diet-induced obesity and lipid metabolism

**Figure 5:**
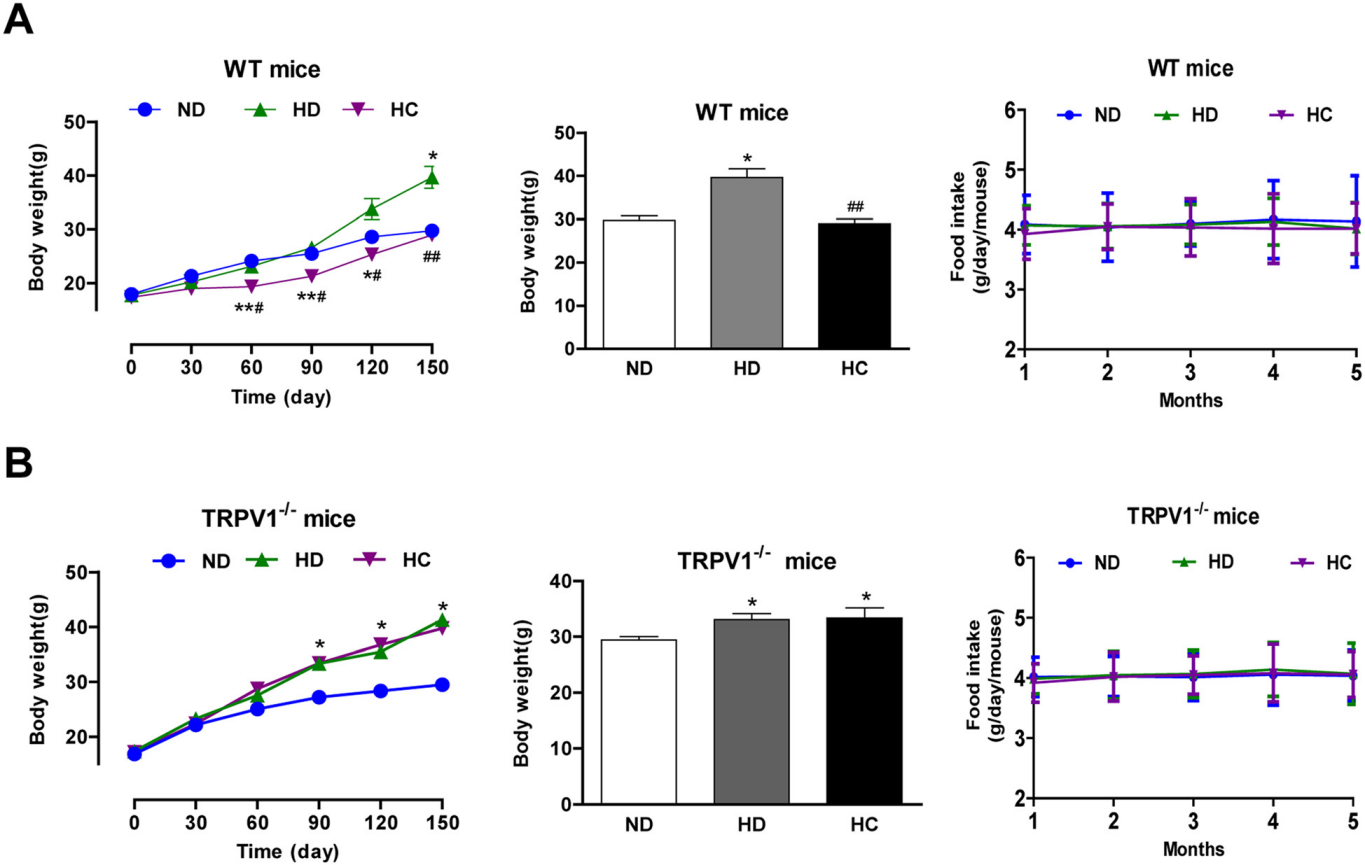
**TRPV1 activation by dietary capsaicin improves high-fat diet-induced obesity. A** and **B**. Body weight gain and food intake of WT **A** and TRPV1^−/−^ **B** mice fed with ND, HD and HC. The values are expressed as the mean ± SEM for 15 mice. *P < 0.05, **P < 0.01 vs. ND, ^##^P < 0.01 vs. HD.

The body weights of WT and TRPV1^-/-^ mice fed a high-fat diet (HD) were significantly higher than those of the ND group mice after a 2-month intervention. Because chronic activation of TRPV1 by dietary capsaicin promoted lipolysis of visceral fat, we sought to determine whether TRPV1 activation by capsaicin reduced the body weight, food intake, serum TG, total cholesterol (TC), low-density lipoprotein cholesterol (LDL-C) and high-density lipoprotein cholesterol (HDL-C). We found that chronic dietary capsaicin significantly reduced the body weights of WT mice fed a high-fat diet after 5 months, but that the effect was absent in TRPV1^-/-^ mice (Figure 5A and B). The food intake was no difference between each group both in WT and TRPV1^-/-^ mice. Dietary capsaicin also lowered serum lipid levels in WT mice fed a high-fat diet (Table 1). Thus, long-term dietary capsaicin lowers the body weight and serum lipid levels in mice fed a high-fat diet.

**Table 1:** **Biochemical characteristics of WT and TRPV1** ^**−/−**^ **mice**

	**WT mice**			**TRPV1** ^**-/-**^ **mice**		
	**ND(n = 15)**	**HD(n = 15)**	**HC(n = 15)**	**ND(n = 15)**	**HD(n = 15)**	**HC(n = 15)**
TG (mmol/l)	0.71 ± 0.04	1.04 ± 0.10**	0.70 ± 0.04^#^	0.68 ± 0.13	0.91 ± 0.06*	0.87 ± 0.07*
TC (mmol/l)	2.10 ± 0.15	3.16 ± 0.19**	2.76 ± 0.48^#^	1.97 ± 0.21	2.46 ± 0.15*	2.26 ± 0.18*
LDL-C (mmol/l)	0.84 ± 0.07	1.14 ± 0.04**	0.97 ± 0.03^#^	0.86 ± 0.11	1.01 ± 0.05*	0.97 ± 0.02*
HDL-C (mmol/l)	0.81 ± 0.09	0.85 ± 0.09	0.65 ± 0.18	0.79 ± 0.38	0.80 ± 0.10	0.82 ± 0.11

Data are mean ± SEM for 15 mice. *P < 0.05, **P < 0.01 vs. WT ND, ^#^P < 0.05 vs. WT HD.

## Discussion

In this study, we found that functional TRPV1 co-expressed with Cx43 in both visceral adipose tissue and 3T3-L1 preadipocytes. TRPV1 activation by capsaicin increased intracellular calcium levels, affected the function of Cx43, and further promoted lipolysis in 3T3-L1 preadipocytes. Long-term chronic capsaicin increased the expression of p-CaM, Cx43, CaMKII, PPARδ and HSL in visceral adipose tissues from WT and db/db mice and obese humans. These results suggest that long-term dietary capsaicin promotes visceral fat remodeling and prevents high-fat-diet-induced obesity in mice by increasing the Ca^2+^ influx through the TRPV1 channels mediated by Cx43.

Capsaicin is the major pungent ingredient in chili peppers and is a highly selective agonist for the TRPV1 channels expressed in sensory neurons and adipose tissues [8,9]. TRPV1 is a non-selective cation channel that is permeable to Na^+^, Ca^2+^ and Mg^2+^ and mediates transmembrane cation flow after stimulated activation. TRPV1 is activated via phosphorylation by protein kinases, the calcium and calmodulin- dependent protein kinase II (CaMKII kinase) and cleavage of phosphatidylinositol 4, 5-bisphosphate (PIP2) by phospholipase C [35]. The N-terminus of TRPV1 has several phosphorylation sites for protein kinases which aid in its activation whereas TRPV1 desensitization results from its dephosphorylation by phosphatises [36]. Capsaicin activates TRPV1 resulting from extracellular calcium dependent conformational changes in the receptor protein, ultimately closing the channel pore. Extracellular calcium influx triggers the release of the intracellular calcium pool and enhances the intracellular free calcium concentration [37]. The movement of calcium ions through the cell membrane into the cell is considered a marker of the intracellular signaling pathway, and a series of basic activities affecting cellular energy metabolism is initiated. Intracellular calcium ions are considered important molecules in hypertension, insulin resistance, and obesity. Our previous study has shown that chronic activation of endothelial TRPV1 channel, which mediates an increased Ca^2+^ influx and subsequent phosphorylation of PKA and eNOS. TRPV1 activation by capsaicin might protect against hyperglycemia-induced endothelial dysfunction through a mechanism involving the PKA/UCP2 pathway [38].

Moreover, a high-calcium diet can reduce lipid accumulation and promote lipolysis and weight loss [39]. In the early stages of adipocyte differentiation, increasing intracellular free calcium levels can prevent adipocyte differentiation and maturation [40]. Intracellular Ca^2+^ is one of the important signaling molecules of adipogenesis. Our previous studies showed that activation of TRPV1 by dietary capsaicin prevents obesity by inhibiting adipocyte differentiation and maturation with an increase in the induction of Ca^2+^ influx [20]. Therefore, intracellular free calcium regulates lipid metabolism and is related to adipocyte differentiation.

Some molecules play roles in lipid metabolism through calcium channels. Connexin is a direct channel regularly present between cells that regulates signal transduction, ions, amino acids, nucleotides, second messengers and other metabolic factors; it allows the passage of molecules with molecular weights less than 1000 Da [41]. Calmodulin (CaM), a cytosolic Ca^2+^ binding protein, is a single-chain acidic protein with a weight of 16700 Da. CaM has four regions with similar structures that can be combined with calcium ions per molecule. When the intracellular calcium concentration transiently increases to 10 ~ 100 times the extracellular calcium concentration, CaM is activated after binding to calcium ions and then combines with enzymes. The intracellular calcium concentration, regulated by calcium ions, is important for biochemical reactions. CaM is proposed to play a role in the Ca^2+^-induced uncoupling of gap junctions because CaM inhibitors were shown to prevent this response [42]. CaM binding sites were recently identified in the cytoplasmic loops of at least three α-subfamily connexins (Cx43, Cx44, and Cx50).

Cx43 gap junctions are gated by a Ca^2+^/CaM-dependent mechanism involving the carboxyl-terminal quarter of the connexin cytoplasmic loop domain [43]. The amino acid residues in the Cx43- carboxyl terminus are targets of multifunctional protein kinases such as Ca^2+^/CaM protein kinase II (CaMKII), an enzyme known to play critical roles in Ca^2+^ homeostasis, transcription, apoptosis, and ischemic heart disease. The activity of CaMKII is important for regulating Cx43 in normal and diseased tissues [44]. Cx43 is the main junction protein in the differentiation and proliferation of adipocytes. The inhibition of Cx43 expression and function induces osteoblast trans-differentiation into adipocytes and allows muscle cells to differentiate into adipocytes [45]. Furthermore, the inhibition of gap junctions promotes osteoblast cell differentiation into adipocytes and the expression of lipoprotein lipase and PPARγ2, resulting in lipid droplet accumulation [46]. In the process of 3T3-L1 preadipocyte differentiation, the connexin inhibitor, 18α-GA, or Cx43 RNA interference inhibits 3T3-L1 cell differentiation and reduces the expression levels of PPARγ2 and GLUT4 in mature adipocytes [23]. Therefore, gap junctions play important roles in adipocyte differentiation and lipid deposition.

Our results showed that the activation of a TRPV1-induced calcium influx was reduced in the case of Cx43 inhibition. Previous studies showed that the Ca^2+^ status between cells is affected by adjusting the gap junction [47]; connexin expression affects the transmission of information between cells, including the transfer of Ca^2+^ [48]. Calcium influx and calcium ion permeability are closely related given that Ca^2+^-dependent ATP inhibitors reduce the transient influx of Ca^2+^. Others found that in neonatal rat cultured myocytes, dominant-negative (DN) inhibition of Cx43 impaired intercellular coupling and desynchronized Ca^2+^ transients among individual cells [49]. The status of gap junction channels is related to the intracellular pH value, ionic changes, cAMP inhibition, Cx protein phosphorylation and genetic factors. The level of intracellular Ca^2+^ is one of the important factors that affect the permeability of connexin expression. The intracellular Ca^2+^ concentration influences connexin function [50], and intracellular calcium overload in ventricular myocytes affects the coupling function between cells [51]. Thus, Cx43 activity is affected by intracellular free Ca^2+^ and regulates intracellular calcium levels.

Peroxisome proliferator activated receptor (PPAR) δ and hormone sensitive lipase (HSL) play critical roles in energy balance, including TG metabolism, fatty acid handling and storage, and glucose homeostasis; the dysregulation of these processes characterizes obesity, diabetes, and atherosclerosis. PPARs are ligand-activated nuclear receptors that are involved in the transcriptional regulation of energy balance and inflammation. Nutrients such as fatty acids direct cellular biology by inducing specific transcriptional responses in the nucleus. PPARs are involved in a mechanism through which nutrient-driven transcriptional regulation can occur [52]. Recent studies suggest that PPARδ regulates lipid metabolism in adipocytes both in vitro and in vivo. PPARδ therapies have complementary effects in improving lipoprotein subfractions associated with atherogenic dyslipidemia [53]. Pharmacological activation of PPARδ inhibited lipogenesis in high-fat diet-induced diabetic mice [54]. The release of free fatty acids from fat stores requires the enzymatic activity of lipases. Mobilization of fatty acids from TG stores in adipose tissue requires lipolytic enzymes. HSL is the only rate-limiting enzyme in the lipolysis of TG stored in adipocytes, and the classical lipolytic PKA/HSL pathway is activated by isoproterenol, forskolin and IBMX [55]. Genetic activation of HSL in mice decreases the adipose mass and leads to triacylglycerol deposition in multiple tissues. A high-fat diet induces the suppression of HSL and AMPK signaling in lipolysis and the utilization of lipids in visceral and subcutaneous adipocytes, which may play an important role in the defective lipid mobilization and metabolism observed in diet-induced obesity [56]. HSL participates in lipolysis through fatty acid β-oxidation [57], while PPARδ activation can increase the levels of HSL and CPT1 in adipose tissue [58]. Our previous study also showed that chronic dietary capsaicin promotes the lipolysis of fatty liver by up-regulating HSL and PPARδ in WT mice [21]. The present study showed that TRPV1 activation increased the expression levels of Cx43, p-CaM and CaMKII through an increased intracellular free calcium concentration and triggered a series of physiological cellular activities. The up-regulation of p-CaM and CaMKII activated PPARδ and HSL and promoted lipolysis.

Our previous studies showed that capsaicin activates TRPV1 and promotes extracellular Ca^2+^ influx [41,59], whereas the present study showed that TRPV1 activation enhances the function of gap junctions, increases extracellular Ca^2+^ influx and activates Cx43. Therefore, we showed that TRPV1 activation induces an influx of calcium mediated by Cx43, and furthermore that Cx43 influenced intracellular Ca^2+^ levels to promote lipolysis and visceral fat remodeling.

## Conclusions

In conclusion, we provided new evidence that TRPV1 activation by dietary capsaicin promotes visceral fat remodeling through the up-regulation of Cx43, which may represent a novel strategy for the management of obesity. Thus, dietary capsaicin may represent a promising lifestyle intervention in populations that are at high risk for obesity.

## Abbreviations

TRPV1: Transient receptor potential vanilloid 1
Cx43: Connexin43
WT: Wild-type
18α-GA: 18 alpha-glycyrrhetinic acid
GJC: Gap-junctional communication
FFA: Free fatty acids
FRAP: Fluorescence replacement after photobleaching
HD: High-fat diet
TC: Total cholesterol
LDL-C: Low-density lipoprotein cholesterol
HDL-C: High-density lipoprotein cholesterol
CaM: Calmodulin
CaMKII: Ca^2+^/CaM protein kinase II
PPAR: Peroxisome proliferator activated receptor
HSL: hormone sensitive lipase
